# Acetylcholine and necroptosis are players in follicular development in primates

**DOI:** 10.1038/s41598-018-24661-z

**Published:** 2018-04-18

**Authors:** Yongrui Du, Konstantin Bagnjuk, Maralee S. Lawson, Jing Xu, Artur Mayerhofer

**Affiliations:** 1 Division of Reproductive & Developmental Sciences, Oregon National Primate Research Center, Oregon Health & Science University, 505 NW 185th Avenue, Beaverton, Oregon, 97006 USA; 2 Department of Reproductive Medicine, Tianjin Central Hospital of Gynecology Obstetrics, No 156 Nankai Sanma Road, Nankai District, Tianjin, 300100 China; 3 BMC Munich, Cell Biology, Anatomy III, Ludwig-Maximilians-University, Grosshaderner Str. 9, D-82152 Planegg, Martinsried Germany; 4 Department of Obstetrics and Gynecology, School of Medicine, Oregon Health & Science University, 3181 SW Sam Jackson Park Road, Portland, Oregon, 97239 USA

## Abstract

Acetylcholine (ACh) in the ovary and its actions were linked to survival of human granulosa cells *in vitro* and improved fertility of rats *in vivo*. These effects were observed upon experimental blockage of the ACh-degrading enzyme (ACH esterase; ACHE), by Huperzine A. We now studied actions of Huperzine A in a three-dimensional culture of macaque follicles. Because a form of programmed necrotic cell death, necroptosis, was previously identified in human granulosa cells *in vitro*, we also studied actions of necrostatin-1 (necroptosis inhibitor). Blocking the breakdown of ACh by inhibiting ACHE, or interfering with necroptosis, did not improve the overall follicle survival, but promoted the growth of macaque follicles from the secondary to the small antral stage *in vitro*, which was correlated with oocyte development. The results from this translational model imply that ovarian function and fertility in primates may be improved by pharmacological interference with ACHE actions and necroptosis.

## Introduction

Acetylcholine (ACh) is known as an important neurotransmitter of the central and the peripheral nervous systems. Its actions are mediated by nicotinic and muscarinic receptors. In addition, ACh is also produced by non-neuronal cells in various organ systems. Roles of non-neuronal ACh are emerging in the skin, the respiratory system, the cardiovascular system, the immune systems and the reproductive system^1–4^.

In the ovary, granulosa cells are producers and targets of ACh^5,6^. Previous studies, mainly using cultured granulosa cells collected from patients undergoing *in vitro* fertilization, implicated ACh in the regulation of cell viability and proliferation. ACh induced muscarinic receptor-mediated elevations of intracellular Ca^2+^ levels and transcription factor expression, activation of ion channels and breakdown of gap junction communication, which resulted in trophic, growth-promoting actions^4,7–10^. Studies in mice indicated that follicle-stimulating hormone (FSH) stimulated ACh production by granulosa cells^11^. Thus, ACh could participate in mediating FSH-actions in the avascular compartment of the ovary. Studies in the bovine corpus luteum were also in line and supported the trophic action of ACh in the ovary^12^.

In neurons, ACh is cleaved and deactivated by acetylcholinesterase (ACHE). *ACHE* was also expressed by granulosa cells collected from patients undergoing *in vitro* fertilization^13^. Blocking ACHE activity by a potent and selective inhibitor, Huperzine A, consequently enhanced granulosa cell survival in culture^13^. A subsequent systemic study in rats demonstrated that Huperzine A, when applied locally to the ovarian bursa, increased intra-ovarian ACh levels and promoted specifically the growth of preantral follicles to the early antral stage^14^. Furthermore, the treatment significantly enhanced antral follicle maturation, ovulation and fertility outcomes. Since ACHE blockers are commonly used in the treatment of Alzheimer’s disease^15^, they could be explored as agents to facilitate ovarian follicular development via regulating granulosa cell viability and proliferation.

The study mentioned above^13^ also led to the insight that cultured human granulosa cells can die not only by apoptosis, but also by necroptosis, i.e. programed necrotic cell death, which was not previously described in ovarian cells^13,16–19^. Necroptosis involves necrosome assembly, i.e., a cascade of interacting kinases cumulating in the execution of necrotic cell death. Interfering with activities of necrosome components, e.g., by necrostatin-1, inhibited necroptosis in cultured human granulosa cells^13^. While the existence of necroptosis in the rodent ovary remains to be determined, follicular expression of phosphorylated mixed lineage kinase domain-like protein (MLKL), pMLKL(S358) as a necroptosis marker, was detected in the human and nonhuman primate antral follicles by immunohistochemistry^13^. This implies physiological relevance of necroptosis in the primate ovary. It remains to be studied, which factors trigger ovarian necroptosis^19^. Although necroptosis occurred in cultured granulosa cells, there are no insights into its regulation, aside from a peptide derived from a splice variant of *ACHE*, namely “read-through variant” (ACHE-R)^20,21^, which enhanced this process^13^.

Based on the results obtained in human granulosa cells and in the systemic rat study, additional experiments were designed, in which the consequences of (1) pharmacological manipulation of ACh breakdown and (2) interference with necroptosis were studied in nonhuman primate growing follicles from the secondary to the small antral stage *in vitro*.

## Results

### Huperzine A treatment

Under control conditions, *in vitro*-developed macaque antral follicles produced ACh, which was detectable in the culture media. Huperzine A addition during culture increased (*P* < 0.05) media concentrations of ACh compared with the vehicle control group (1.23 ± 0.09 versus 0.93 ± 0.06 µM; n = 26 and 17 follicles from 5 animals, respectively).

The percentage of rhesus macaque follicles that survived relative to the total number cultured was 56 ± 9% in the vehicle control group at culture week 5. Huperzine A addition had no effect on follicle survival after 5 weeks of culture (58 ± 11%) compared with controls.

**Figure 1:**
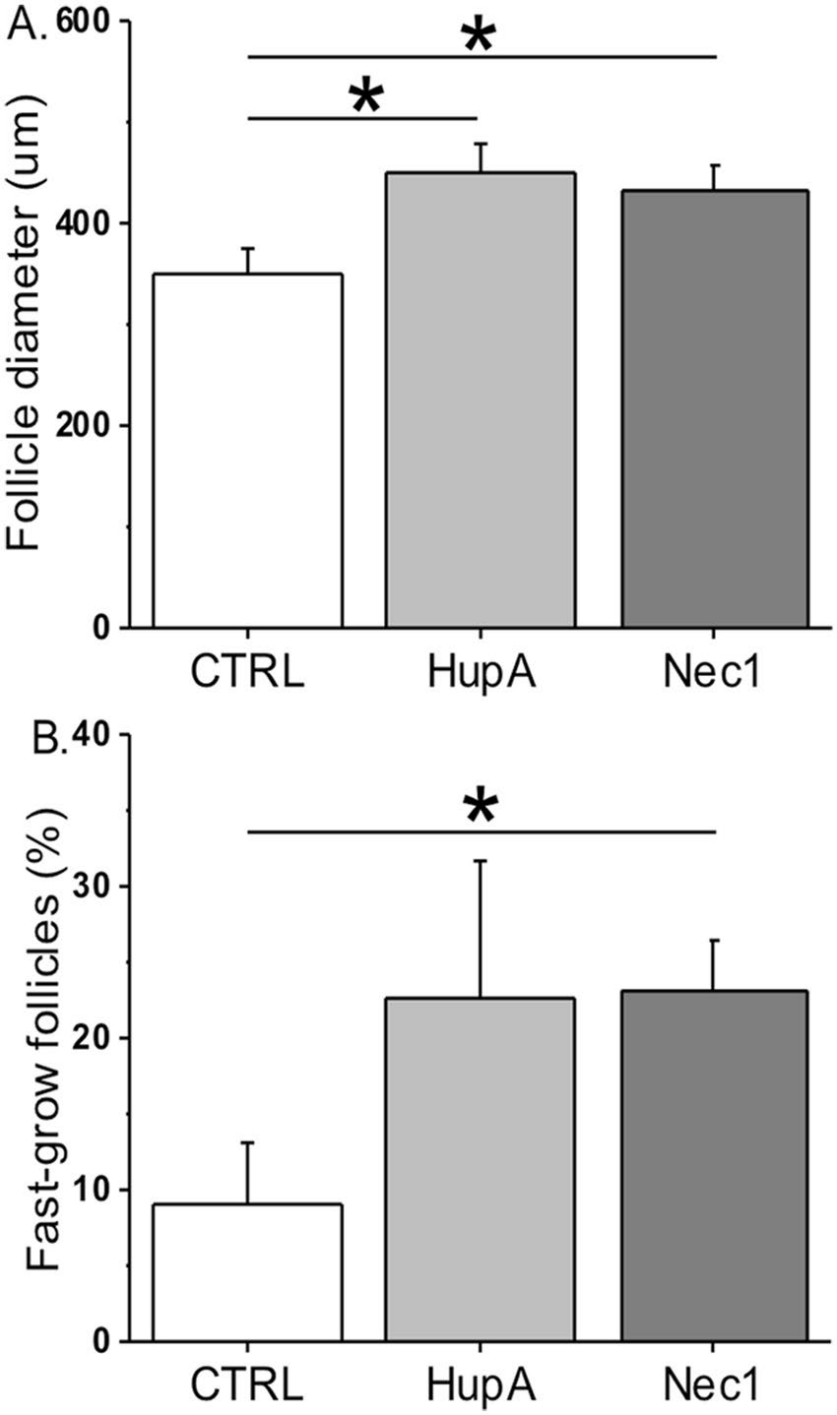
The effects of Huperzine A and necrostatin-1 on rhesus macaque follicle growth after 5 weeks of culture in an alginate matrix. Follicle growth was presented as follicle diameters (**A**) and as percentages of fast-grow follicles (diameter ≥ 500 μm) versus total growing follicles (**B**). CTRL, vehicle control; HupA, Huperzine A addition; Nec1, necrostatin-1 addition. *Significant difference between treatment groups (*P* < 0.05). Data are presented as the mean ± SEM with 18–31 follicles and 5 animals per experimental group.

Rhesus macaque follicles that survive *in vitro* can be divided into distinct cohorts based on their growth by week 5 as previously described^22^. While non-growing follicles remained at the preantral stage throughout 5 weeks of culture, growing follicles formed an antrum at week 3. The percentages of growing versus total surviving follicles were comparable between the vehicle control and Huperzine A group (50 ± 7 versus 73 ± 13%). However, though starting with equivalent sizes in the beginning of culture, growing follicles attained larger (*P* < 0.05) diameters at week 5 of culture in the Huperzine A group compared with those of the vehicle control group (Fig. 1A). Growing follicles with diameters ≥ 500 μm at culture week 5 were termed as fast-grow follicles, as previously described^22^. There were no significant differences between the vehicle control and Huperzine A group on the percentages of fast-grow versus total growing follicles at culture week 5 (Fig. 1B).

**Figure 2:**
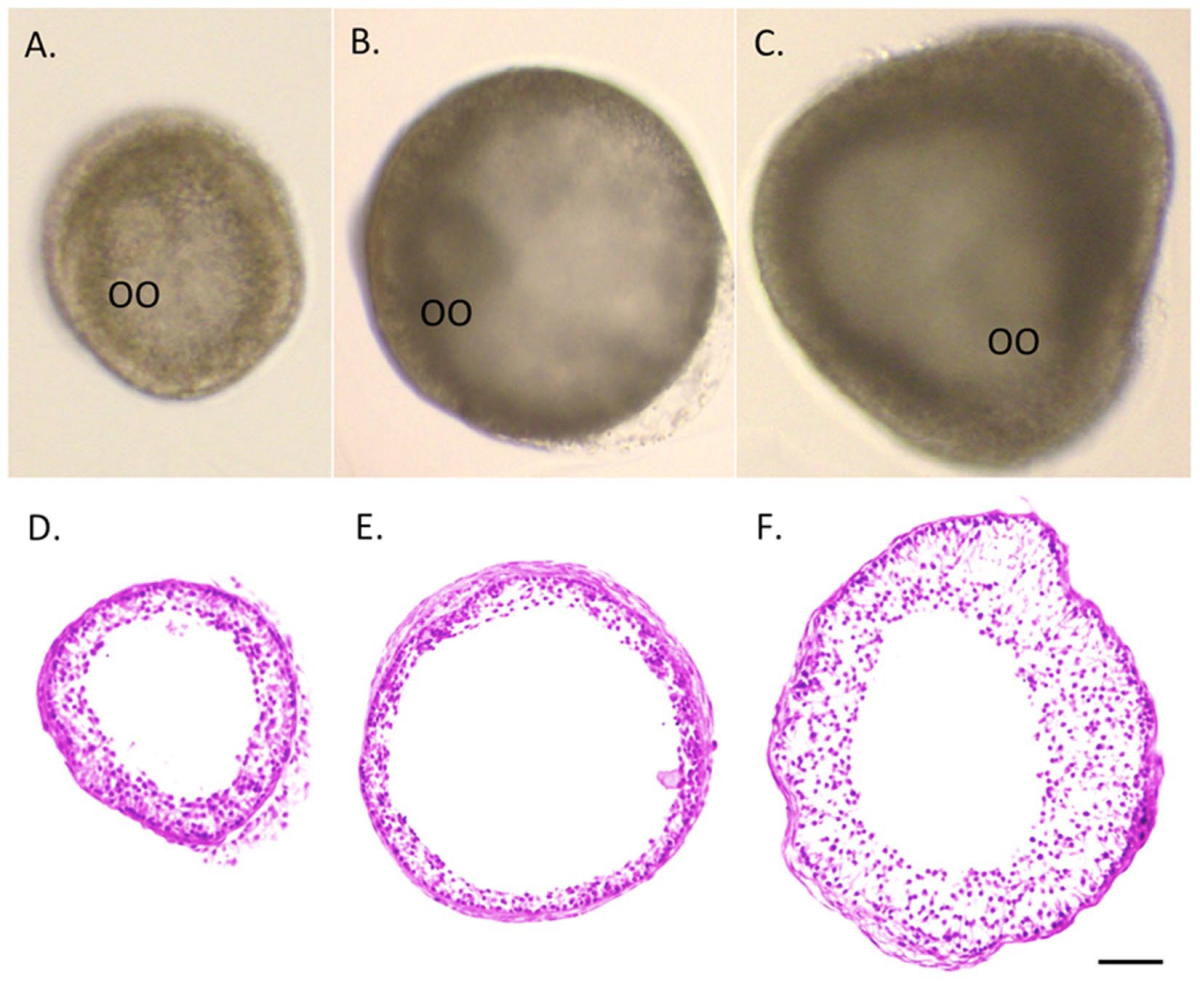
*In vitro*-developed rhesus macaque follicles following 5 weeks of culture in an alginate matrix with exposure of the control vehicle (**A**), Huperzine A (**B**) and necrostatin-1 (**C**) were stained with hematoxylin and eosin, respectively (**D**,**E**,**F**). OO, oocyte. Scale bar = 100 µm.

Following 5 weeks of culture, hematoxylin and eosin staining of follicles (the largest sections of follicles to show the relative follicle diameters and the thickness of follicle walls) from the vehicle control (Fig. 2A and D) and Huperzine A (Fig. 2B and E) groups revealed a morphology similar to that observed in *in vivo*-developed small antral follicles in primates^23^, in terms of a spherical shape with an oocyte and an antral cavity, multiple layers of granulosa cells and an intact basement membrane.

*In vitro*-developed macaque antral follicles produce appreciable amounts of ovarian steroid hormones, including progesterone (P4), androstenedione (A4) and estradiol (E2), into the culture media, as reported previously^22^. Huperzine A addition did not statistically alter media P4, A4 or E2 concentrations produced by growing follicles compared with those of the vehicle controls at culture week 5 (P4: 11 ± 4 versus 8 ± 4 ng/ml; A4: 3 ± 2 versus 5 ± 3 pg/ml; E2: 487 ± 175 versus 289 ± 88 pg/ml).

**Figure 3:**
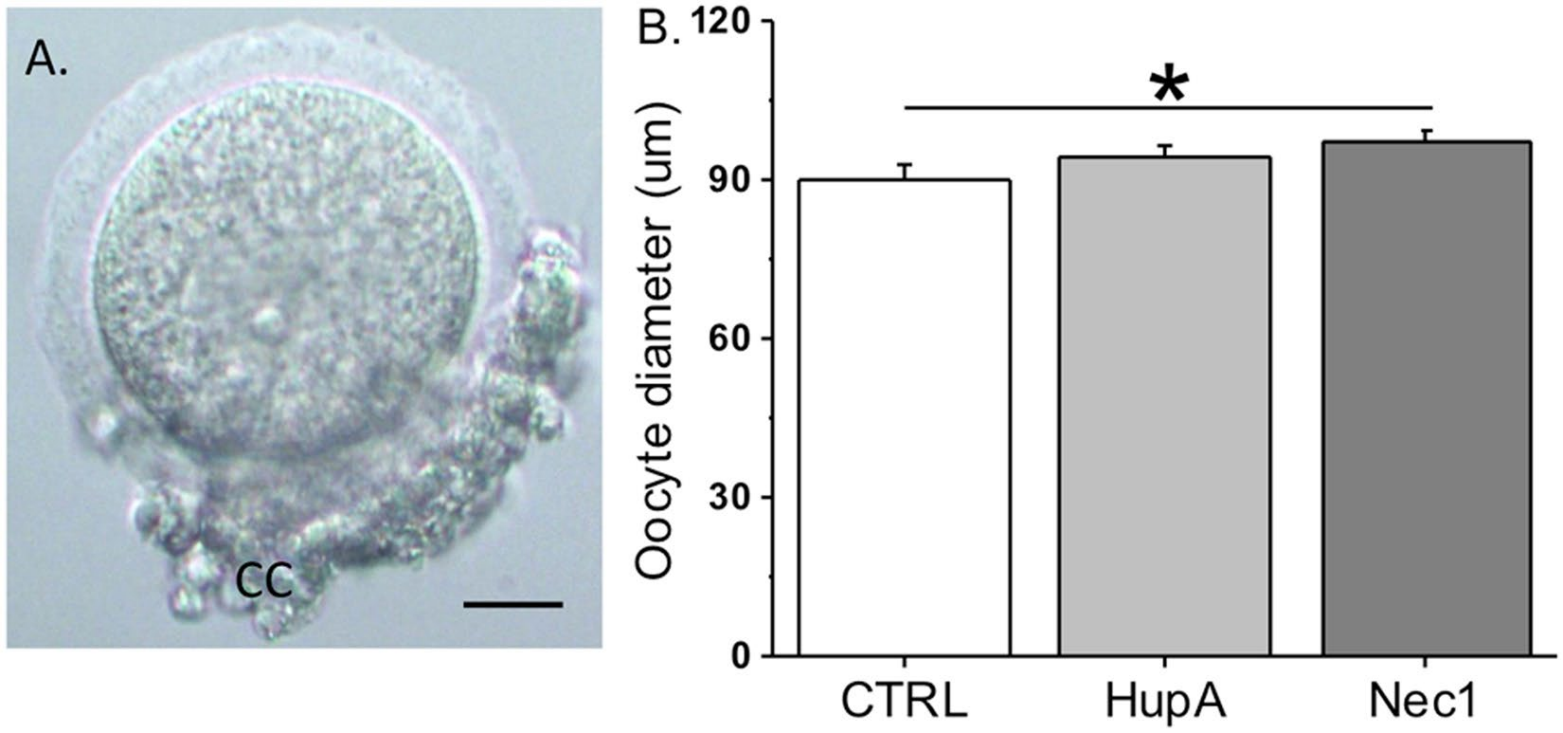
The effects of Huperzine A and necrostatin-1 on oocyte growth in rhesus macaque antral follicles after 5 weeks of culture in an alginate matrix. Oocytes obtained were surrounded by cumulus cells (A; representative from the vehicle control group). Oocyte growth was determined by measuring oocyte diameters. CC, cumulus cells; CTRL, vehicle control; HupA, Huperzine A addition; Nec1, necrostatin-1 addition. *Significant difference between treatment groups (*P* < 0.05). Data are presented as the mean ± SEM with 18–31 oocytes per experimental group. Scale bar = 25 µm.

Healthy, germinal vesicle stage oocytes surrounded by cumulus cells were obtained from *in vitro*-developed macaque antral follicles after 5 weeks of culture in the vehicle control and Huperzine A groups (Fig. 3A). Huperzine A treatment did not alter the oocyte diameters relative to the control group (Fig. 3B).

### Necrostatin-1 treatment

Neither the follicle survival rates nor the percentage of growing versus total surviving follicles at culture week 5 were altered by necrostatin-1 treatment relative to the vehicle control group (survival: 64 ± 10 versus 56 ± 9%; growth: 69 ± 15 versus 50 ± 7%). However, diameters of growing follicles at culture week 5 were larger (*P* < 0.05) in the necrostatin-1 group than those of the vehicle control group (Fig. 1A). In addition, percentages of fast-grow follicles were greater (*P* < 0.05) following necrostatin-1 exposure compared with those of the vehicle controls (Fig. 1B). Hematoxylin and eosin staining at culture week 5 showed that follicles cultured with necrostatin-1 had an extensively developed granulosa layer (Fig. 2C and F) relative to follicles in the vehicle control group (Fig. 2A and D).

There were no statistically significant differences between the vehicle control and necrostatin-1 group on the media concentrations of P4 (8 ± 4 versus 5 ± 2 ng/ml), A4 (5 ± 3 versus 2 ± 2 pg/ml) or E2 (289 ± 88 versus 723 ± 265 pg/ml) produced by growing follicles at culture week 5. *In vitro*-developed macaque antral follicles in the necrostatin-1 group produced healthy, germinal vesicle stage oocytes surrounded by cumulus cells. Compared with the vehicle controls, the oocyte diameters at week 5 increased (*P* < 0.05) in follicles following necrostatin-1 exposure (Fig. 3B).

### Expression of *ACHE* and of necrosome components

**Figure 4:**
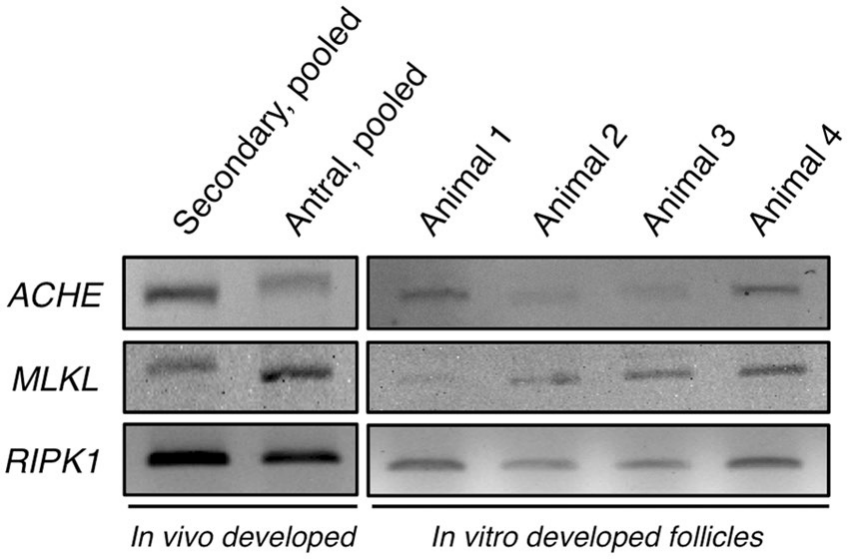
RT-PCR analysis on the expression of acetylcholinesterase (*ACHE*), mixed lineage kinase domain-like protein (*MLKL*), receptor-interacting serine/threonine-protein kinase 1 (*RIPK1*) by *in vivo*-developed secondary and antral follicles pooled from 4 macaques, respectively, as well as by *in vitro*-developed antral follicles (under control culture conditions) from 4 individual macaques. Electrophoresis and Midori Green staining were performed using the same protocol for all gels. Results were cropped and grouped. Original gels are provided in the supplemental dataset.

The mRNA expression of *ACHE* and the necrosome components (*MLKL*, receptor-interacting serine/threonine-protein kinase 1 or *RIPK1*) were detected in *in vivo*-developed macaque secondary and antral follicles (Fig. 4). *In vitro*-developed antral follicles from all 4 macaques expressed *ACHE*, *MLKL*, *RIPK1*, as identified by RT-PCR (Fig. 4). Amplicon identities were confirmed by sequencing.

**Figure 5:**
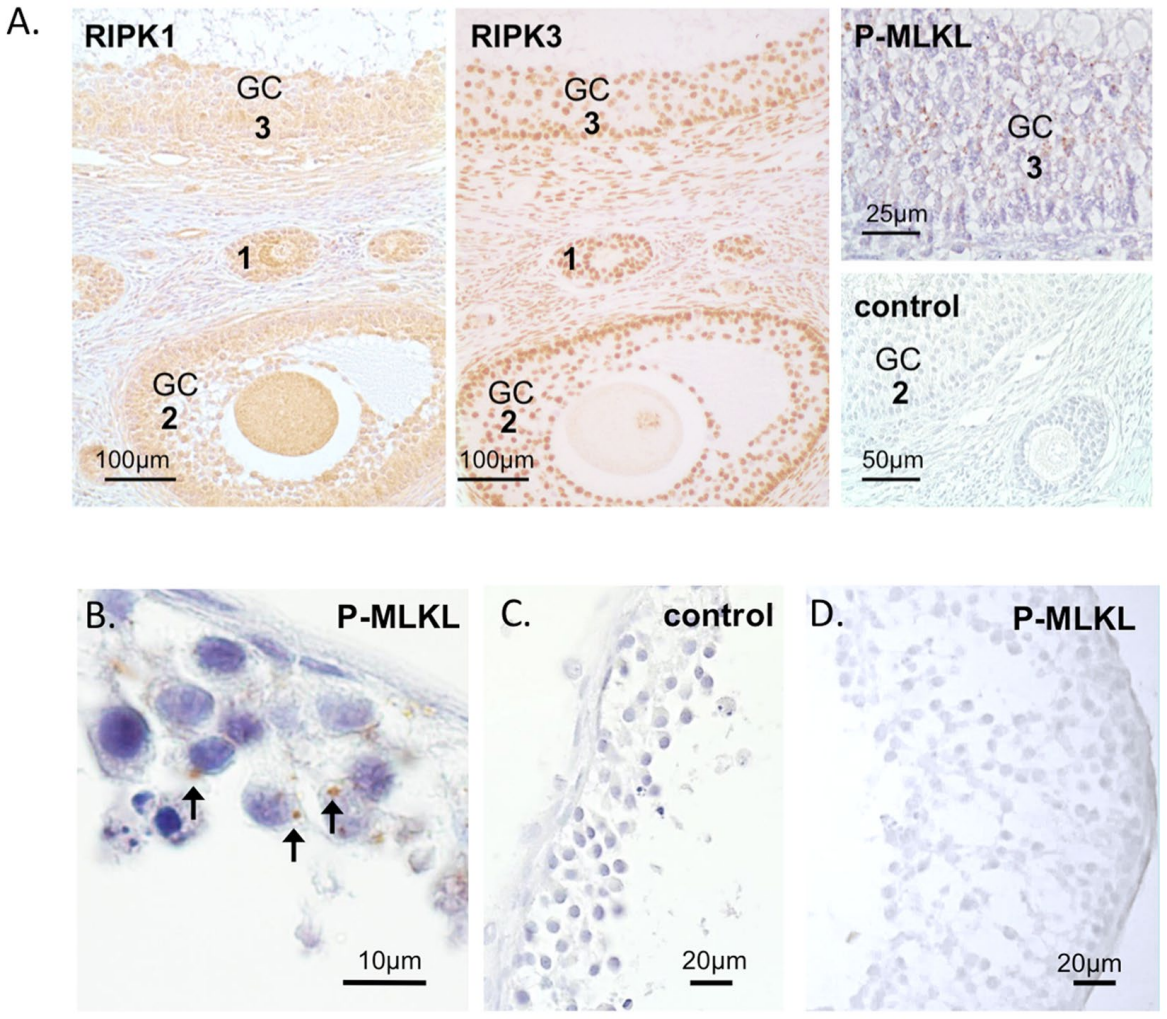
Immunohistochemical detection of necroptosis-related proteins in macaque ovarian follicles. Receptor-interacting serine/threonine-protein kinase 1 (RIPK1) and 3 (RIPK3) were identified in *in vivo*-developed preantral (1), small antral (2) and large antral (3) follicles, mainly in granulosa cells (GC) (**A**). Granulosa cells of some follicles were stained for phosphorylated mixed lineage kinase domain-like protein, pMLKL(S358) (**A**). Positive staining of pMLKL(S358) was also detected in the granulosa cells of *in vitro*-developed small antral follicles cultured under control conditions (**B**; arrows), but not in the negative controls (**A**,**C**) or follicles cultured with necrostatin-1 (**D**). Counterstaining was performed using haematoxylin.

In addition, RIPK1 and RIPK3 proteins were readily detected in the preantral and antral follicles, mainly in granulosa cells, of macaque ovaries by immunohistochemistry (Fig. 5A). Granulosa cells of some *in vivo*-developed macaque follicles were stained for pMLKL(S358) (Fig. 5A). Positive immunostaining of pMLKL(S358) was also detected in the cytoplasm of granulosa cells in *in vitro*-developed antral follicles from all 4 macaques (Fig. 5B). No pMLKL(S358)-positive staining was evident in negative control sections (Fig. 5A and C) or *in vitro*-developed antral follicles cultured with necrostatin-1 (Fig. 5D).

## Discussion

The present study utilized a three-dimensional follicle culture system^22^ to explore roles of ACh and necroptosis during primate follicular development. Pharmacological inhibition of ACh-breakdown was achieved by ACHE-blocker Huperzine A. Necroptosis was inhibited by intercepting RIPK1 actions using necrostatin-1. Both approaches enhanced follicular development from the secondary to the small antral stage *in vitro*. The results of the present study performed in a translational model reveal previously unknown roles for ACh/ACHE and necroptosis in follicular development, and indicate that pharmacological agents, which target these processes, may be suitable to enhance follicular development.

ACh production and some of its actions were previously identified in granulosa cells of the ovarian follicle. ACh acted via muscarinic receptors, which were detected in macaque and human ovarian follicles^4^. Downstream actions studied in human granulosa cells included among others breakdown of gap junctions and increasing proliferation^7,8^. Further studies in granulosa cells obtained from patients undergoing *in vitro* fertilization demonstrated the positive impact of ACh on cell viability in culture^13^. The addition of Huperzine A, which blocks ACHE actions^24^, generated similar outcomes^13^. In a follow-up systemic study in rats, Huperzine A administration to the ovarian bursa elevated ovarian ACh levels, specifically enhanced the growth of preantral follicles, improved ovulation, and increased overall fertility^14^. When the same concentration of Huperzine A was used for macaque follicle culture in the present study, it promoted follicle growth from the secondary to the small antral stage, as indicated by larger follicle diameters, implying a trophic action of ACh in granulosa cells. ACh levels produced by *in vitro*-developed follicles increased following Huperzine A treatment, as demonstrated in the current study, and higher bioavailable ACh stimulated granulosa cell proliferation and viability in macaque follicles, which is consistent with previous studies.

The previous study in rats^14^ showed that Huperzine A treatment increased the number of small antral follicles *in vivo*. However, in our current study, blocking ACHE actions did not increase the overall survival of macaque follicles developed *in vitro*, which could be due to the fact that follicle survival involves both granulosa cell and oocyte viability. Oocytes may have an important role in regulating follicular development^25^. In this context, expression of receptors for ACh in rhesus macaque preovulatory follicle-derived oocytes^7^ indicates that the oocyte could be a direct target of ACh. Future studies are warranted to determine whether oocytes from preantral and small antral follicles are affected by ACh and what the outcomes are.

Once ovarian follicles start to grow, they either reach the preovulatory stage or undergo atresia. Apoptosis of oocytes and granulosa cells has been intensively studied and is suggested to be the underlying mechanism of follicular atresia throughout species^26,27^. Recently, necroptosis was described in cultured human granulosa cells^13^, which could be intercepted by drugs targeting the necrosome^16^. In human granulosa cells, the RIPK1 inhibitor necrostatin-1 and the MLKL blocker necrosulfonamide reduced necroptosis significantly^13^. A specific marker pMLKL(S358) was detected in human and macaque granulosa and luteal cells, which provided first evidence of necroptosis in the primate ovary^13^.

Consistently, the present study demonstrated expression of the necrosome components *MLKL* and *RIPK1* in macaque follicles developed *in vivo* and *in vitro*. RIKP1, RIPK3 and pMLKL(S358) proteins were also detected in macaque ovarian follicles. The presence of pMLKL(S358) clearly indicated ongoing necroptosis in macaque follicles. To further explore involvement of necroptosis in follicular development, necrostatin-1 was introduced into the follicle culture system with the same concentration as what was used in human granulosa cell culture^13^. Macaque secondary follicles cultured in the presence of necrostatin-1 grew larger than the control follicles as indicated by increased follicle diameters, greater percentages of fast-grow follicles and well-developed granulosa layers. Therefore, besides apoptosis, necroptosis appears to be an important additional mechanism in regulating follicular cell death, at least in the primate ovary.

Although diameters of *in vitro*-developed antral follicles increased following either Huperzine A or necrostatin-1 exposure, more fast-grow follicles were obtained only from the necrostatin-1 group containing oocytes with larger diameters. It could be due to a suboptimal dose of Huperzine A employed, the greater impact of necroptosis in follicular growth, in general. These points, which are the heart of the question how follicular growth is regulated, require additional studies. Growth of follicles is also reflected by active E2 production by well-developed granulosa cells, specifically in the necrostatin-1-treated follicles, though the differences in media E2 levels did not reach statistical significance. Results from the current study suggest that inhibition of necroptosis has the potential to promote primate follicular development, which may be used to improve outcomes of *in vitro* follicle maturation protocols.

Several ACHE-blockers are used clinically for the treatment of Alzheimer’s disease^15,28,29^. Necroptosis-blockers are being developed and tested for treatment of various medical conditions^15^. It appears conceivable that these agents could also be used to manipulate follicular development, either by enhancing granulosa cell proliferation or by interfering with granulosa cell necroptosis. Studies are warranted to explore their effectiveness in treatment of ovarian dysfunction.

It is not clear whether the cholinergic system in the ovary^6^ is affected by circulating ACHE or the related enzyme, butyrylcholine-esterase (BCHE). Both enzymes break down ACh and were active in human follicular fluid^13^. ACHE and BCHE increase in the circulation with age in women^30^. Hence, it seems that changes in the circulating levels of these enzymes could be superimposing factors affecting the fate of ovarian follicles by lowering available ACh. The age-related decline of the functional ovarian reserve is thought to be a consequence of follicular atresia, which ultimately leads to depletion of the ovarian follicle pool, and hence, menopause^26,27,31^. Studies are now warranted to explore the involvement of ACh/ACHE and necroptosis in the process of follicular atresia.

In summary, both Huperzine-A and necrostatin-1 promoted overall follicular development during encapsulated three-dimensional culture in rhesus macaques, presumably by fostering granulosa cell proliferation (actions of elevated ACh) and limiting granulosa cell necroptosis (actions of interference with RIPK1). The results reveal, for the first time, the importance of local ACh and necroptosis in the regulation of primate folliculogenesis, which supports the potential of pharmacological interference of ACHE actions and necroptosis as novel approaches to improve ovarian functions in women.

## Methods

### Animal use and ovary collection

The general care and housing of rhesus macaques (*Macaca mulatta*) were provided by the Division of Comparative Medicine, Oregon National Primate Research Center (ONPRC), Oregon Health & Science University, as previously described^22^. Animals were pair-caged in a temperature-controlled (22 °C), light-regulated (12 L: 12D) room. The diet consisted of Purina monkey chow (Ralston-Purina, Richmond, IN, USA) and was provided twice a day supplemented with fresh fruit or vegetables once a day. Water was provided *ad libitum*. Animals were treated according to the National Institutes of Health’s Guide for the Care and Use of Laboratory Animals. Protocols were approved by the ONPRC Institutional Animal Care and Use Committee^22^.

Ovaries were collected from 5 animals at necropsy (8–14 year old) by the Pathology Services Unit, via the ONPRC Tissue Distribution Program. Euthanasia was not performed for the current study, but was due to health issues unrelated to reproductive health. Ovaries were immediately transferred into HEPES-buffered holding media (Cooper Surgical, Inc., Trumbull, CT, USA) and kept at 37 °C for follicle isolation^32^.

### Follicle isolation, encapsulation and culture

The process of follicle isolation, encapsulation and culture was previously reported^22^. Briefly, the ovarian cortex was cut into 1 × 1 × 1 mm cubes. Follicles were mechanically isolated using 31-gauge needles. Secondary follicles (diameter 125–225 μm) met criteria for encapsulation if they exhibited an intact basement membrane, 2–4 layers of granulosa cells and a healthy centrally located oocyte.

Follicles were individually transferred into 5 µl 0.25% (w/v) sterile sodium alginate (FMC BioPolymers, Philadelphia, PA, USA)-PBS (137 mM NaCl, 10 mM phosphate, 2.7 mM KCl, Invitrogen, Carlsbad, CA, USA). The droplets were gelled in 50 mM CaCl_2_, 140 mM NaCl, 10 mM HEPES solution (pH 7.2). Each encapsulated follicle was placed in individual wells of 48-well plates containing 300 µl alpha minimum essential medium (Invitrogen) containing 6% (v/v) human serum protein supplement (Cooper Surgical, Inc.), 0.5 mg/ml bovine fetuin, 5 µg/ml insulin, 5 µg/ml transferrin, 5 ng/ml sodium selenite (Sigma-Aldrich, St Louis, MO, USA), and 3 ng/ml recombinant FSH (NV Organon/Merck Sharp & Dohme, Oss, Netherlands)^22^.

Follicles from each of the five animals were randomly assigned to 3 experimental groups (12 follicles/monkey/group): (a) vehicle control (0.025% ethanol), (b) 10 µM Huperzine A (42643; Sigma-Aldrich), and (c) 20 µM necrostatin-1 (sc-200142; Santa Cruz Biotechnology, Inc., Santa Cruz, CA, USA). Follicles were cultured at 37 °C in a 5% O_2_ environment (in 6% CO_2_/89% N_2_) for 5 weeks. Media (150 µl) was collected and replaced every other day, and stored at −20 °C^22^.

### Follicle survival and growth

Follicle survival, growth and antrum formation were assessed weekly using an Olympus CK-40 inverted microscope and an Olympus DP11 digital camera (Olympus Imaging America Inc., Center Valley, PA, USA), as described previously^22^. Follicle growth was determined by measuring the distance from the outer layer of cells at the widest diameter and then the diameter perpendicular to the first measurement by the same individual. The mean of the two values determined the follicle’s overall diameter. The measurements were performed using ImageJ 1.6.0 software (National Institutes of Health, Bethesda, MD, USA). Follicles were considered atretic if the oocyte was dark or not surrounded by a layer of granulosa cells, the granulosa cells appeared dark or fragmented, or the follicle diameter decreased.

### Follicle histology

Randomly selected *in vitro*-developed antral follicles from all three groups were harvested at culture week 5 and fixed in in 4% paraformaldehyde-PBS solution for 3 hours at room temperature. Follicles were embedded in HistoGel (Thermo Scientific, Kalamazoo, MI, USA) before being dehydrated in ascending concentrations of ethanol (70–100%) and embedded in paraffin. Five micrometer sections were cut by the Histopathology-Morphology Research Core at ONPRC, and stained with hematoxylin and eosin as previously described^22^.

### Culture media assays

To determine the efficiency of Huperzine A in blocking ACHE actions in cultured follicles, media samples from the control and the Huperzine A group were analyzed for ACh concentrations using the Amplex Red Acetylcholine/Acetylcholinesterase Assay Kit (A12217; Molecular Probes, Inc., Eugene, OR, USA) according to the manufacturer’s instruction, as described previously^33^.

In order to assess steroidogenesis in cultured follicles, media samples collected from each culture group were analyzed for P4, A4 and E2 concentrations by the Endocrine Technologies Core at ONPRC. P4 and E2 were assayed to determine granulosa cell steroidogenic function using an Immulite 2000, a chemiluminescence-based automatic platform (Siemens Healthcare Diagnostics, Deerfield, IL, USA)^22^. A4 was measured by ELISA to determine thecal cell steroidogenic function using an AA E-1000 kit (Rocky Mountain Diagnostics, Inc., Colorado Springs, CO, USA) according to the manufacturer’s instruction^22^.

### Oocyte evaluation

Oocyte evaluations were performed on a 37 °C warming plate, as previously described^22^. Briefly, the cumulus-oocyte complex was dissected out of the follicle in Tyrode’s albumin lactate pyruvate (TALP)-HEPES-BSA (0.3% v/v) medium provided by the Assisted Reproductive Technologies Core at ONPRC. Oocytes were then transferred to TALP medium and photographed. Oocyte diameters (excluding the zona pellucida) and conditions were assessed using the same camera and software, as described above.

### Expression of *ACHE* and necrosome components in macaque follicles

This retrospective study included follicles obtained from animals (n = 4) reported in previous research^34^. Briefly, *in vivo*-developed secondary (30 follicles/monkey) and antral (10 follicles/monkey) follicles were isolated from the cortex and the medulla region of macaque ovaries, respectively, and pooled. *In vitro*-developed antral follicles were collected at the end of culture under control conditions and pooled (10 follicles/monkey). Total RNA was extracted from each follicle pool for reverse transcription, as previously described^34^. Oligonucleotide primers for PCR (Table 1) were designed using Primer3^35,36^ and synthesized by metabion international AG (Planegg, Germany). PCR was performed to examine expression of *ACHE*, *MLKL* and *RIPK1*, as described previously^13^. PCR products were sequenced by GATC Biotech AG (Konstanz, Germany) and analyzed using BLAST^37^.

**Table 1:** Information about oligonucleotid primers used for RT-PCR.

Target gene	Direction	Sequence	*Macaca mulatta* fitness	Accession number
*MLKL*	Forward	5′- TAC AGT CAG CAG AGT GCA GG -3′	85%	*H. sapiens* NM_152649.2 *M.Mulatta* XM_015126624.1
	Reverse	5′- ACC GTT TGT GGA TGA CCT GG -3′	95%	
*RIPK1*	Forward	5′- TGG GCG TCA TCA TAG AGG AAG -3′	100%	*H. sapiens* NM_003804 *M.Mulatta* XM_015135439.1
	Reverse	5′- CGC CTT TTC CAT GTA AGT AGC A -3′	100%	
*ACHE*	Forward	5′- TTC CTC AGT GAC ACC CCA GA -3′	100%	*H. sapiens* NM_000665.4 *M. Mulatta* NM_001128088.2
	Reverse	5′- GGG GAG AAG AGA GGG GTT AC -3′	100%	

Note that primers were designed using *Homo sapiens* RNA sequences. Except *MLKL* forward (85%) and *MLKL* reverse (95%), all primers are 100% identical to *Macaca mulatta* sequences. Sequences of PCR products obtained matched *Macaca mulatta* sequences as confirmed upon sequencing.

Consecutive sections from paraffin-embedded rhesus macaque ovaries (n = 3) obtained from previous studies^11,13^ were used for immunohistochemistry with antibodies detecting RIPK1 (HPA015257; Sigma-Aldrich), RIPK3 (HPA055087; Sigma-Aldrich) and pMLKL(S358) (ab187091; Abcam, Cambridge, UK), as previously described^13^. Randomly selected paraffin embedded antral follicles (4 follicles from 4 macaques), developed under control culture conditions^34^, were obtained for sectioning and immunohistochemistry for pMLKL(S358). To determine the efficiency of necrostatin-1 in blocking necroptosis in cultured follicles, *in vitro*-developed antral follicles harvested from the necrostatin-1 group at week 5 were also stained for pMLKL(S358). Controls were performed with the omission of antibodies or using non-immune serum.

### Statistical analysis

Statistical analysis was performed using SigmaPlot 11 software (SPSS, Inc., Chicago, IL, USA). Because follicles from each animal were randomly distributed into the culture groups, the Wilcoxon signed-rank test was used to evaluate differences in follicle survival and percentage of fast-grow follicles, with five individual animals in each experimental group. One-way analysis of variance (ANOVA), followed by the Student-Newman-Keuls post hoc test, was used to analyze diameters of follicles and oocytes, as well as media hormone concentrations, with total follicle numbers indicated in the figure legends which represent follicles obtained from five individual animals. Media ACh concentrations were analyzed using a Student’s *t*-test. Differences were considered significant at *P* < 0.05 and values are presented as mean ± SEM.

### Data availability

Data generated during this study are included in this published article and supplementary files. The original raw datasets generated during the current study are available from the corresponding author on reasonable request.
